# Study on the protective effect of *Aronia melanocarpa* extract on type 2 diabetes by regulating glucose and lipid metabolism through intestinal flora

**DOI:** 10.1002/fsn3.4378

**Published:** 2024-07-31

**Authors:** Dan Song, Cheng Fang

**Affiliations:** ^1^ Second Affiliated Hospital Heilongjiang University of Chinese Medicine Harbin P. R. China; ^2^ Drug Safety Evaluation Center Heilongjiang University of Chinese Medicine Harbin P. R. China

**Keywords:** *Aronia melanocarpa* extract, gut flora, glucose and lipid metabolism, type 2 diabetes

## Abstract

*Aronia melanocarpa*, a plant rich in anthocyanins, has been studied for its potential to regulate blood sugar and blood lipids, although the specific mechanism is not yet understood. This research aims to identify the differential bacterial flora and elucidate the mechanism by which it improves glucose and lipid metabolism disorders through 16S rDNA gene sequencing. The study reveals the protective effect of *Aronia melanocarpa* extract (AME) on liver damage in type 2 diabetic rats. Experimental results demonstrate that AME can effectively modulate the abundance of intestinal flora, reduce colon tissue damage, enhance the weight of diabetic rats, and lower levels of fasting blood sugar, low‐density lipoprotein (LDL), and triglycerides (TG). Additionally, liver morphology analysis shows that AME can effectively mitigate liver tissue structural damage in type 2 diabetic rats. In conclusion, AME regulates glucose and lipid metabolism by influencing intestinal flora, ultimately regulating glucose and lipid metabolism in type 2 diabetic rats.

## Graphical abstract

*Aronia melanocarpa* has been studied for its potential to regulate blood sugar and blood lipids, although the specific mechanism is not yet understood. This research aims to identify the differential bacterial flora and elucidate the mechanism by which it improves glucose and lipid metabolism disorders through 16S rDNA gene sequencing. Experimental results demonstrate that *Aronia melanocarpa* extract (AME) regulates glucose and lipid metabolism by influencing intestinal flora, ultimately regulating glucose and lipid metabolism in type 2 diabetic rats.

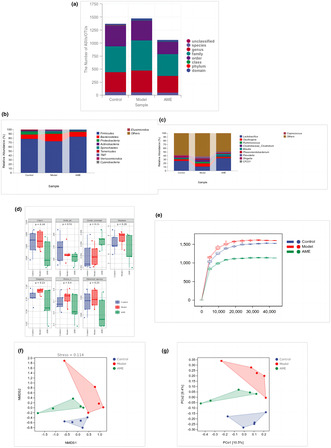

## 1 INTRODUCTION

Type 2 diabetes (T2D) is a complicated metabolic disorder that makes up around 90% of the 537 million worldwide diabetes cases and is on the rise on a global scale (Ahmad et al., 2022). The liver plays a central role in maintaining systemic glucose and lipid homeostasis (Rui, 2014). In hepatocytes, glucose is metabolized through glycolysis, and the byproducts of glycolysis are used to synthesize fatty acids (Harada et al., 2007). Fatty acids, along with triglycerides (TG), phospholipids, and cholesterol esters, can either be stored in lipid droplets or released into the bloodstream (Agius, 2008). Additionally, the liver plays a crucial role in regulating glucose levels by carrying out processes such as glycogenolysis and glycogenesis (Koliaki & Roden, 2013). Impairment of liver function can disrupt glucose and lipid metabolism, thereby accelerating the progression of T2D.

Various pharmacological properties have been discovered in plant products rich in anthocyanins, such as anti‐inflammatory, anti‐tumor, and antioxidant effects. These properties have been scientifically proven to reduce hyperglycemia‐induced oxidative stress and its complications (Liu et al., 2010; Zafra‐Stone et al., 2007; Zhang et al., 2011). *Aronia melanocarpa* is known to have a high content of anthocyanin, which is its main active component (Kähkönen et al., 1999). Studies have shown that consuming *A. melanocarpa* juice has a positive impact on postprandial glucose levels in healthy individuals (Yamane et al., 2017). Furthermore, both the extract and juice of *A. melanocarpa* have been found to regulate blood glucose and blood lipid levels in streptozocin (STZ)‐induced diabetic rats (Maslov et al., 2002; Valcheva‐Kuzmanova et al., 2007). Clinical experiments have also demonstrated that *A. melanocarpa* juice can effectively lower fasting blood glucose levels in patients with type 2 diabetes (Kulling & Rawel, 2008; Simeonov et al., 2002).

Intestinal microflora plays a crucial role in regulating the host's physiological metabolism. The imbalance of intestinal microflora has been found to be associated with the development of diabetes mellitus. Patients with diabetes exhibit a lower bacterial diversity in their intestinal flora, and significant changes in the microbial population can influence immune tolerance and enhance the integrity of the intestinal barrier (Qin et al., 2012). Disruptions in the gut microbiota can lead to damage in intestinal epithelial cells, compromising the integrity of the intestinal barrier. This can result in bacterial endotoxin leakage, systemic inflammation, and disturbances in glucose metabolism (Li et al., 2017). Moreover, damage to the gut lining may trigger T cells that react to islet cells in diabetic individuals, leading to the progression of autoimmune diabetes. Reestablishing a balanced intestinal barrier via fecal microbiota transplant (FMT) could potentially postpone the initiation of diabetes (Sorini et al., 2019). These findings suggest a potential connection between intestinal microflora, the intestinal barrier, and glucose homeostasis.

The objective of this research was to evaluate the impact of systemic injury on diabetic mice through the assessment of body weight, blood glucose levels, and blood lipid profiles. Furthermore, the study observed the morphological changes in the liver and intestinal mucosa of diabetic rats. Additionally, the study discussed the relationship between abnormal glucose and lipid metabolism and intestinal microflora in diabetes. The findings of our study offer a valuable new approach to enhance the intestinal barrier and address intestinal dysbiosis. *Aronia melanocarpa* extracts (AME) show potential as a treatment for diabetes.

## 2 MATERIALS AND METHODS

### 2.1 Determination of anthocyanin in AME

In this experiment, we selected cyanidin‐3‐*O*‐glucoside as the control and AME as the test material. We used a Waters liquid chromatograph 2695‐UV2998 with a Diamonsil 5MC18(2)250 nm × 4.6 mm column. The flow rate was 1 mL/min, and the column temperature was set at 30°C. The sample size was 20 mL, and the volume ratio of water to methanol in the mobile phase was 75:25. The column temperature was further adjusted to 40°C, and the detection wavelength was set at 280 nm.

### 2.2 Experimental animals and design

Male Sprague–Dawley rats (180 ± 20 g) from the Laboratory Animal Center, Heilongjiang University of Chinese Medicine (Harbin, China) were acclimatized for one week. During this period, the rats were housed in a room maintained at a temperature of 23 ± 3°C and subjected to a 12‐h light/dark cycle. The animal care and treatment protocols were approved by the Ethics Committee of Heilongjiang University of Chinese Medicine. To induce diabetes, the rats were fed a high‐fat diet (basic diet +10% lard +20% sucrose +2.5% cholesterol +1% sodium cholate) for 28 days. After a 12‐h fasting period (with free access to water), each rat was intraperitoneally injected with streptozocin (40 mg/kg; Sigma, St. Louis, MO), while the control rats received an injection of buffer only. After 72 h, blood samples were collected from the rats' tail veins to measure fasting blood glucose (FBG, fasting 12 h before blood collection) levels and determine the presence of diabetes (FBG ≥11.1 mmol/L). Group I served as the control group and received only vehicle injections. The diabetic rats were randomly divided into four groups, with eight rats in each group. Group II served as the model group and received vehicle injections. Groups III and IV were administered AME at doses of 100 mg/kg and 400 mg/kg, respectively. Group V received standard metformin at a dose of 200 mg/kg.

### 2.3 Collection and preparation of biological samples

At the conclusion of the experiment, blood was gathered from the rats' abdominal main vein, followed by centrifugation of the samples at room temperature at a rate of 3500 rpm for 10 min. A 50 mL quantity of serum was then obtained. Using a biochemical analyzer, the levels of total cholesterol (CHO), high‐density lipoprotein (HDL), low‐density lipoprotein (LDL), and triglyceride (TG) were assessed. The intestinal contents from the cecum (2 g) were preserved in a sterile cryopreserved tube and promptly frozen in liquid nitrogen. Subsequently, each set of five samples was subjected to high‐throughput sequencing of 16S rRNA.

### 2.4 Analysis of intestinal flora

The gut microbiota was extracted and detected using some modifications. Briefly, total microbial DNA was extracted from the collected cecal contents using a DNA extraction kit. The 16S rRNA V3‐V4 hypervariable region was amplified using the primer sequences 338F (5’‐ACTCCTACGGGGGGCAGG‐3′) and 806R (5’‐GACTACHVGGGTWTCTAAT‐3′). The PCR products were recovered using 2% (w/v) agarose gel electrophoresis and the AxyPrep DNA gel extraction kit.

### 2.5 HE stains

After fixing the liver and colon in a formalin solution, small pieces of fixed liver tissue were flushed with running water for 2 h. Following the washing process, the liver tissues were dehydrated using 70%, 80%, 90%, and 95% anhydrous ethanol. Subsequently, they were soaked in a xylene solution, immersed in paraffin, and embedded. The slides were prepared by cutting the embedded tissues into slices, which were then subjected to ethanol and xylene gradients. Hematoxylin and eosin staining was performed sequentially. Finally, the slides were soaked in ethanol and xylene, and subsequently dried.

### 2.6 Statistical analysis

Analysis of the data was conducted using GraphPad Prism 8 software from GraphPad Software, Inc. located in San Diego, CA, USA. Data are shown as mean ± standard deviation. Differences between multiple groups were assessed using one‐way ANOVA. Levene's test was used to verify variance homogeneity. LSD (assuming equal variance) or Kruskal–Wallis H test (not assuming equal variance), and post hoc tests were used to determine differences between groups (*p* < .05). The alpha value was set to 0.05, and a logarithmic score of linear discriminant analysis (LDA) ≥ 2.0 was used as the threshold.

## 3 RESULTS AND DISCUSSION

### 3.1 Anthocyanin content in AME

**FIGURE 1:**
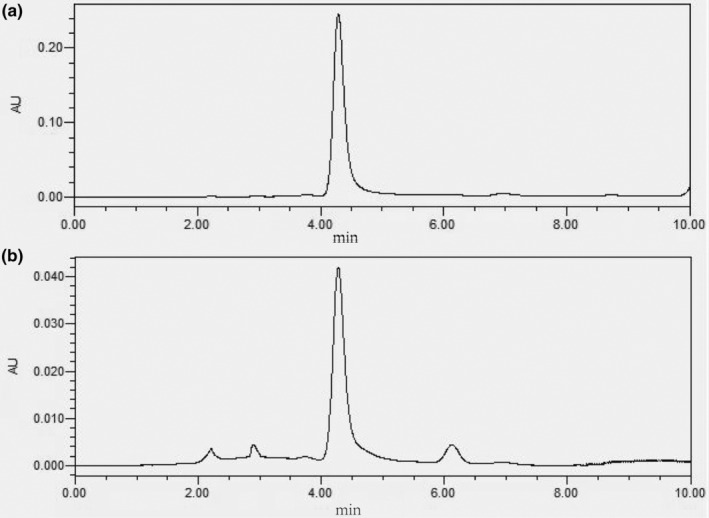
AME determination of cyanidin‐3‐*O*‐glucoside by HPLC. (a) Chromatogram of cyanidin‐3‐*O*‐glucoside, (b) AME chromatogram.

To demonstrate the abundance of anthocyanins in AME, we determined the content of cyanidin‐3‐*O*‐glucoside. The sample concentration was set at 20 mg/mL, and the peak area of the sample was measured to be 13,171,000. The content of anthocyanin was found to be 5 mg/mL, accounting for 25% (Figure 1).

### 3.2 Effects of AME on body weight, blood glucose, and blood lipid in diabetic rats

**FIGURE 2:**
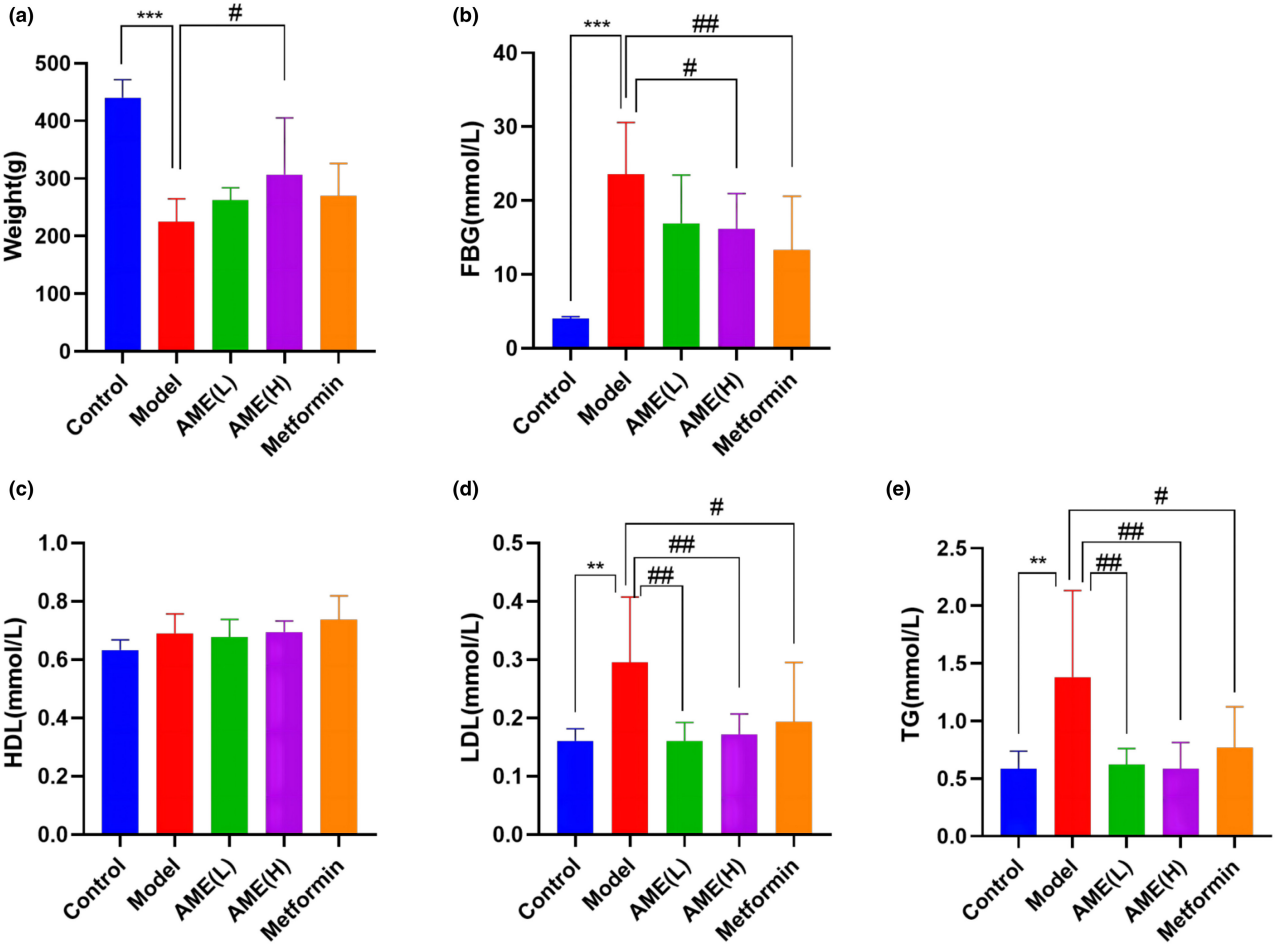
Effects of AME treatment on body weight, blood glucose, and blood lipid in diabetic rates. (a) effects of AME treatment on body weight, (b) effects of AME treatment on FBG, (c) effects of AME treatment on HDL, (d) effects of AME treatment on LDL, (e) effects of AME treatment on TG. ^##^ *p* < .01, ^#^ *p* < .05, compared to the control; ***p < .001, **p < .01, compared to the model.

To investigate the potential benefits of AME treatment on glucose and lipid metabolism in T2D rats, we conducted measurements of body weight, blood glucose, and blood lipid levels (HDL/LDL/TG) (Figure 2). The rats in the model group exhibited lower body weight (*p* < .001), higher blood glucose (*p* < .001), and elevated HDL and TG levels (*p* < .01), indicating abnormal glucose and lipid functions. However, compared to the model group, AME (L) administration resulted in a decrease in LDL/TG levels (*p* < .01), an increase in body weight (*p* < .05), a reduction in blood glucose (*p* < .05), LDL and TG levels (*p* < .01), and a decrease in FBG levels (*p* < .01), LDL and TG levels (*p* < .05) in the metformin group. These findings suggest that AME (h) has the potential to improve blood glucose and blood lipid levels.

### 3.3 Effect of AME treatment on liver pathology in diabetic rats

**FIGURE 3:**
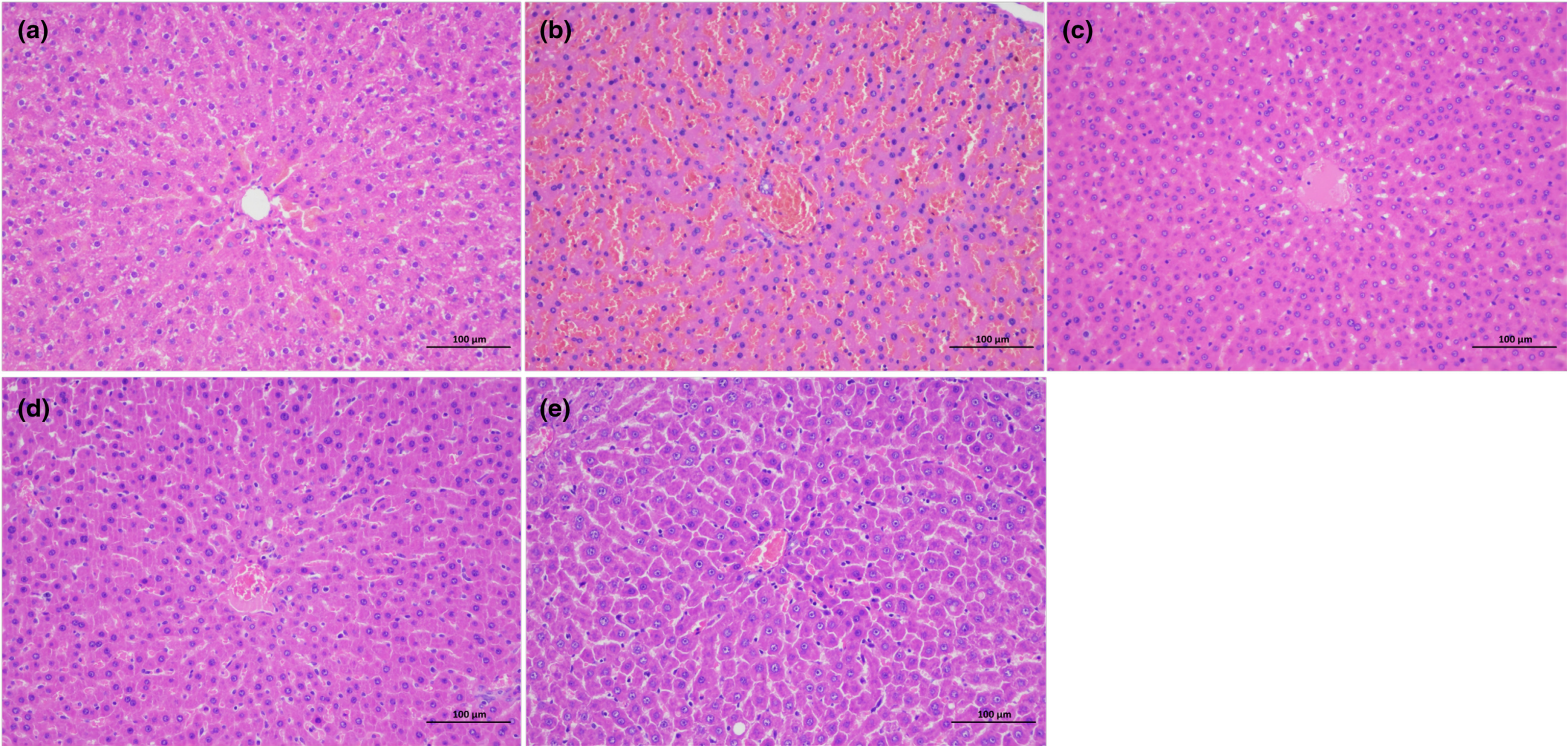
Representative photomicrographs of H&E in liver tissue of different diabetic groups, magnification: 200×. (a) Control, (b) model, (c) metformin, (d) AME(L), (e) AME(H).

The liver tissues of rats in each group were stained with HE (Figure 3). The liver of the model group and metformin group appeared dark yellow and larger in size compared to the blank group. The color of the AME low‐dose group and high‐dose group was more similar to the blank group, with the AME high‐dose group still having a larger volume than the blank group. The results of HE staining showed that compared with the blank group, the hepatic cords in the model group were disordered, with darker nuclei and some ruptured hepatocytes. In the metformin group, the arrangement of hepatic cords improved, with even nuclear staining, although some cells were still broken and more fat vacuoles were observed. The arrangement of hepatic cords in the AME low‐dose group was better than that in the model group. In the AME high‐dose group, the hepatic cords were arranged normally, the morphology of hepatocytes was normal, nuclear staining was even, and the fat vacuoles were significantly reduced.

### 3.4 Composition analysis and diversity of intestinal flora

**FIGURE 4:**
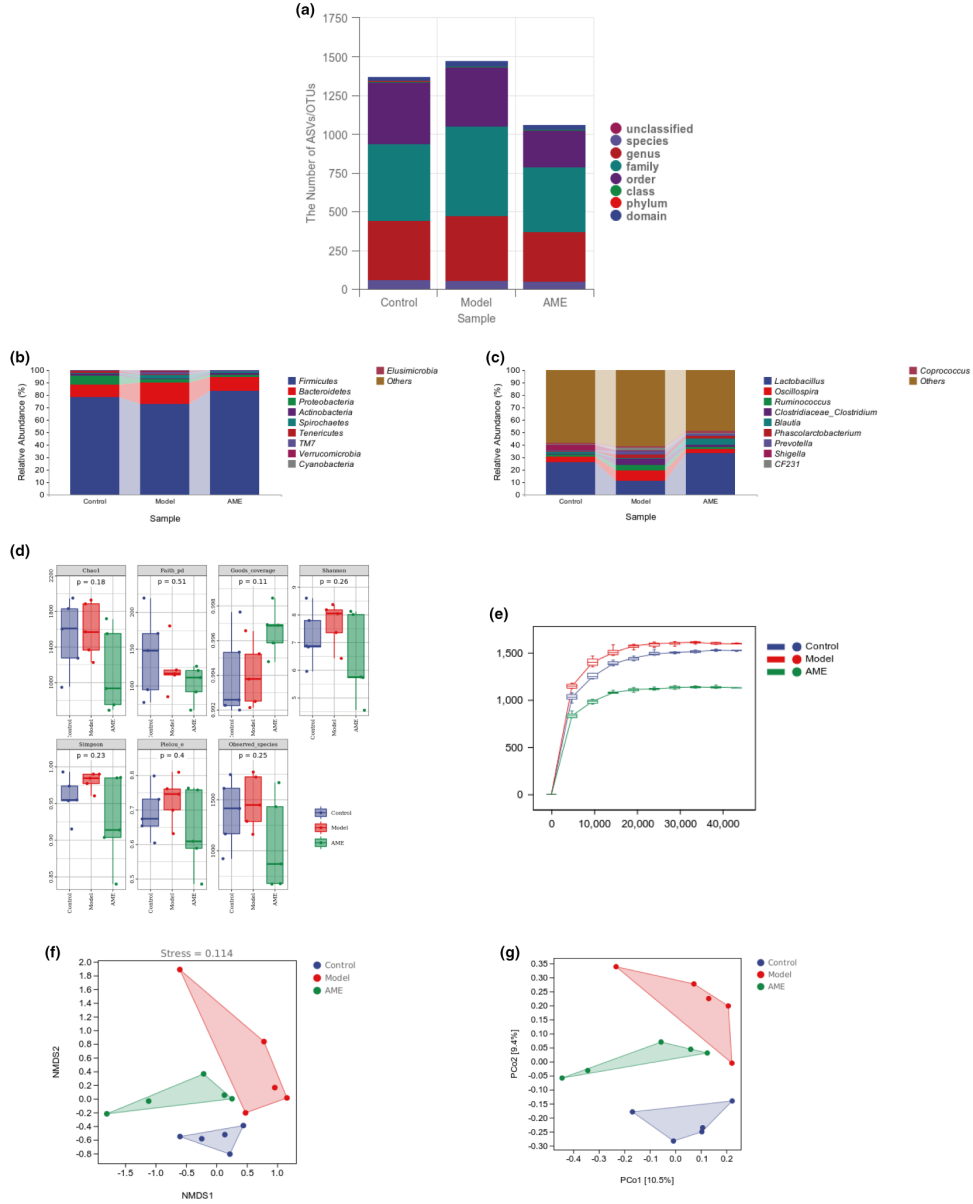
Effect of AME treatment on the structure and diversity of intestinal microflora in diabetic rats. (a) Annotated statistics of species taxonomy. (b) Species taxonomic phyla level composition. (c) The taxonomic genera of species are composed horizontally. (d) Alpha diversity index. (e) The sparse curve. (f) PCOA analysis. (g) NMDS analysis.

**FIGURE 5:**
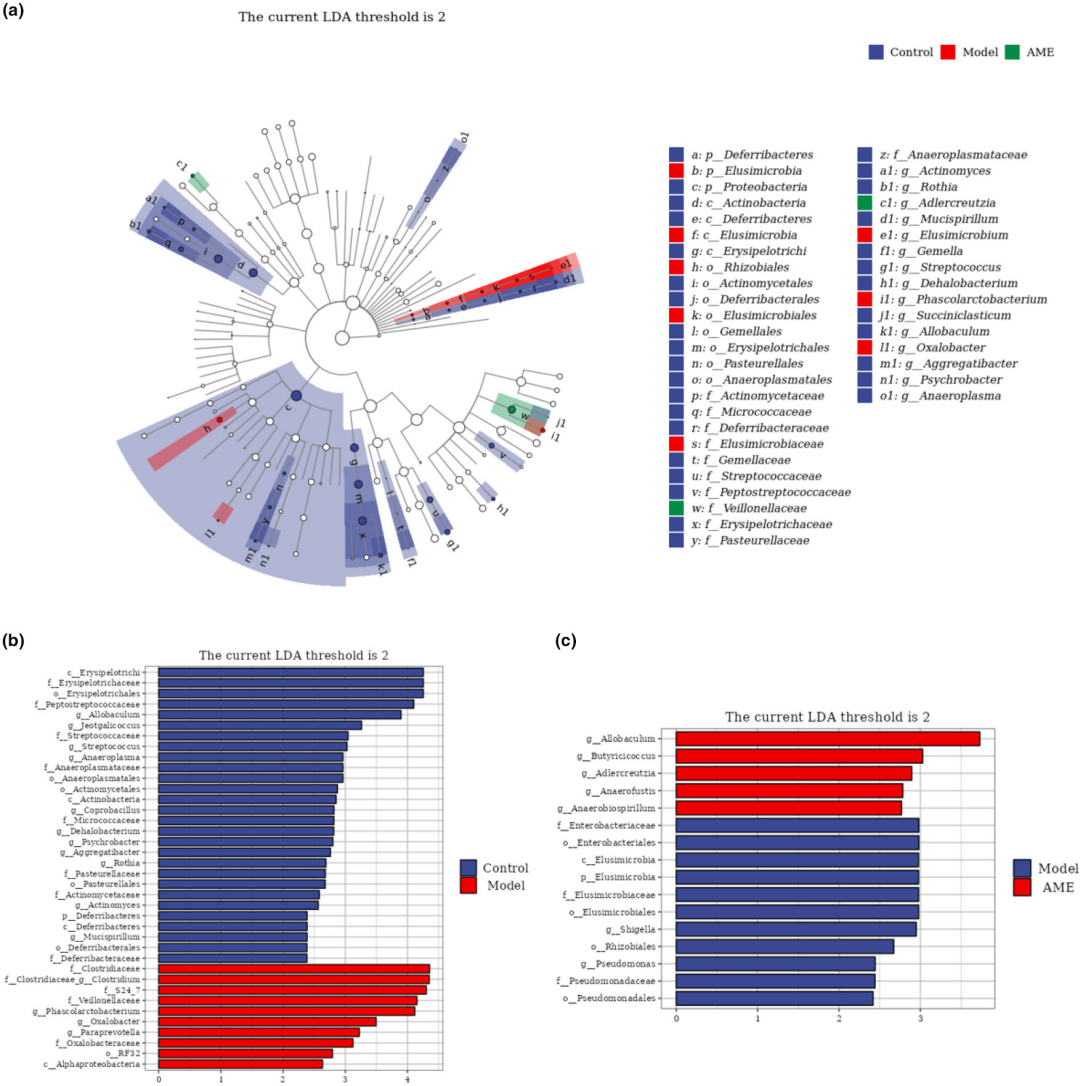
Linear discriminant analysis effect size (LEfSe) integrated with Linear discriminant analysis (LDA) scores recognized differently adjacent tax associated with AME treatment. (a) Cladogram shows the physiological distribution of differential biomarkers. (b) Differential biomarkers between blank and model groups. (c) Differential biomarkers for model and AME groups. The criterion for significant difference is LDA scores >2.

The structure of the intestinal microflora is closely associated with the occurrence and development of diabetes mellitus. In order to demonstrate the ability of AME to alter the intestinal flora of diabetic rats, we conducted an analysis of the intestinal flora in the high‐dose AME group, control group, and model group (Figures 4 and 5). Figure 4a provides a classification of the three groups, allowing for a direct comparison of the number of flora units and the taxonomic status between the blank group, the model group, and the AME group. It is evident from the graph that the resolution of species annotation is higher. Figure 4b,c represents the phylum and genus levels, respectively. The top ten phyla observed were Firmicutes, Bacteroidetes, Actinobacteria, Spirochaetes, TM7, Verrucomicobia, Cyanobacteria, and Elusimicrobia. Among the top ten genera, Lactobacillus, Oscillospira, Ruminococcus, Clostridiaceae_Clostridium, Blautia, Phascolarctobacterium, Prevotella, and CF231 exhibited a significant response following AME treatment.

#### 3.4.1 Results of alpha diversity analysis

Figure 4d displays the alpha diversity index, which comprises various indices such as the Chao1 index, Faith‐pd index, Good‐coverage index, Shannon index, Simpson index, Pielou‐e index, and Observed‐species index. There were no significant differences in the Alpha diversity indices among the blank group, the model group, and the AME group (*p* > .05). In Figure 4e, the horizontal coordinate represents the depth of sample leveling, while the vertical coordinate represents the median value of the alpha diversity index calculated 10 times. The boxplot indicates that the curve tends to flatten, suggesting that the samples from the blank group, model group, and AME group adequately reflect the diversity of the current samples. Increasing the sequencing depth does not lead to the detection of a large number of new ASV/OTUs.

#### 3.4.2 Beta diversity analysis results

PCoA projects the sample distance matrix and expands it in a low‐dimensional space. The distance between samples, as determined by the Jaccard distance algorithm, indicates the dissimilarity between them. In Figure 4f, the results showed a clear and significant difference in distance between the model group and the blank group, indicating a noticeable change in the bacterial group composition of the model group compared to the blank group. However, after administration of AME, the distance between the AME group and the model group completely distinguished the two, showing that the microbial community structure of the AME group was superior to that of the model group. Moreover, the high dose of AME was found to better regulate the microbial community composition of diabetic rats, as depicted in Figure 4g. NMDS, which employs a hierarchical ordering, demonstrates that the closer (farther) the distance between two points, the smaller (larger) the difference in the microbial community between the samples. In this case, the blank group and the model group are the farthest apart, indicating a significant difference in the community structure between them. However, the AME group approached the blank group after AME administration, suggesting that the bacterial community composition of diabetic rats tended to normalize.

#### 3.4.3 LEfSe analysis

LEfSe analysis allows for the direct performance of differential analysis on all classification levels simultaneously, in order to identify iconic species at different levels. The differential species between the blank group and the model group include c‐Erysipelotrichi, f‐Erysipelotrichaceae, o‐Erysipelotrichales, f‐Peptostreptococcaceae, g‐Allobaculum, g‐Jeotgalicoccus, f‐Streptococcaceae, g‐Streptococcus, g‐Anaeroplasma, f‐Anaeroplasmataceae, o‐Anaeroplasmatales, o‐Actinomycetales, c‐Actinobacteria, g‐Coprobacillus, f‐Micrococcaceae, g‐Dehalobacterium, g‐Psychrobacter, g‐Aggregatibacter, g‐Rothia, f‐Pasteurellaceae, o‐Pasteurellales, f‐Actinomycetaceae, g‐Actinomyces, p‐Deferribacteres, c‐Deferribacteres, g‐Mucispirillum, o‐Deferribacterales, f‐Deferribacteraceae, f‐Clostridiaceae, f‐Clostridiaceae‐g‐Clostridium, f‐S24‐7, f‐Veillonellaceae, g‐Phascolarctobacterium, g‐Oxalobacter, g‐Paraprevotella, f‐Oxalobacteraceae, o‐RF32, and c‐Alphaproteobacteria. The different species between the model group and the AME group are, respectively, g‐Allobaculum, g‐Butyricicoccus, g‐Adlercreutzia, g‐Anaerofustis, g‐Anaerobiospirillum, f‐Enterobacteriaceae, o‐Enterobacteriales, c‐Elusimicrobia, p‐Elusimicrobia, f‐Elusimicrobiaceae, o‐Elusimicrobiales, g‐Shigella, o‐Rhizobiales, g‐Pseudomonas, f‐Pseudomonadaceae, and o‐Pseudomonadales. Notably, g‐Allobaculum exhibits significant differences between the blank group and the model group, as well as between the model group and the AME group. The callback effect of AME on g‐Allobaculum is particularly significant. For more details, refer to Figure 4.

### 3.5 Effect of AME on the morphology of intestinal mucosa in diabetic rats

**FIGURE 6:**
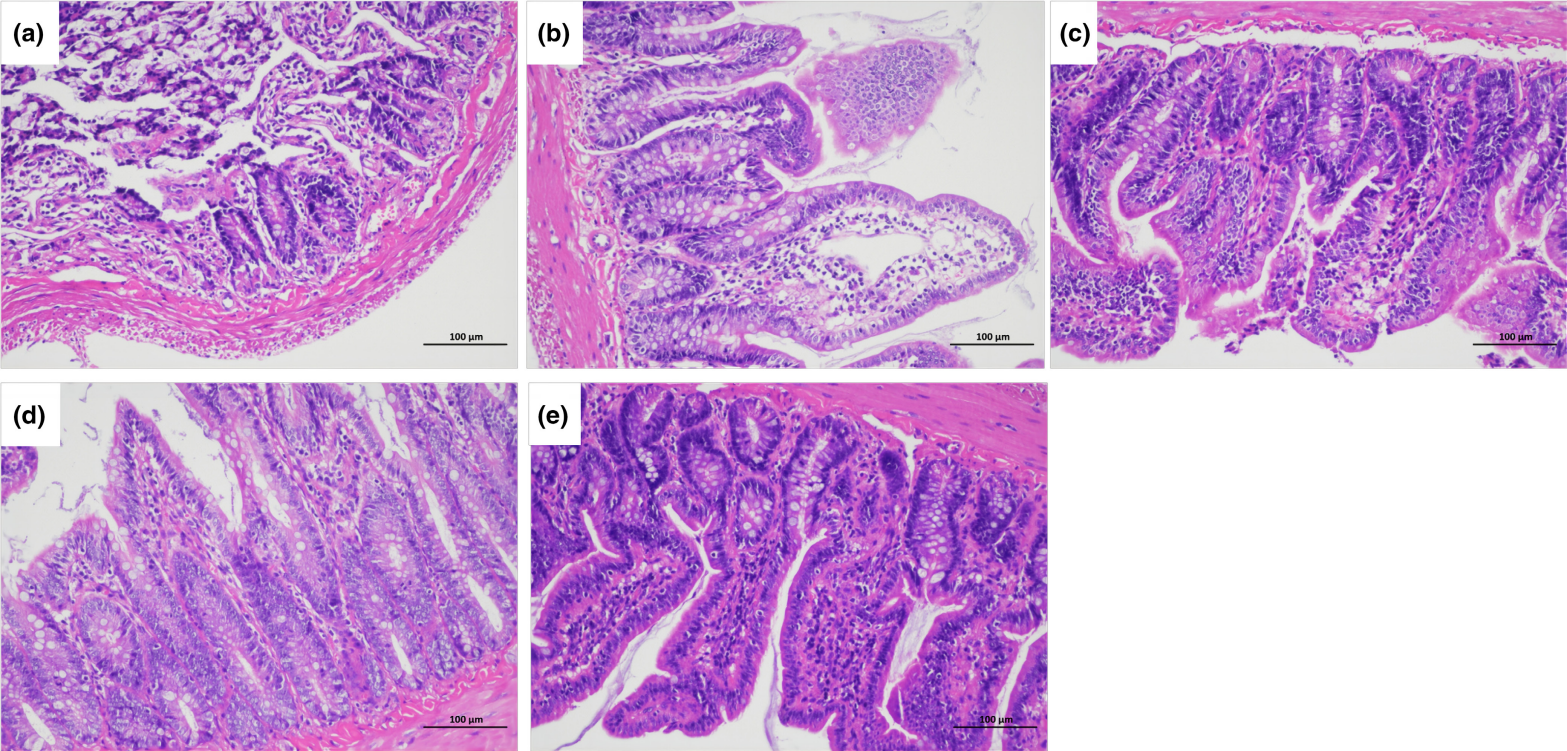
Representative photomicrographs of H & E in the colon of different diabetic groups, magnification: 200×. (a) control, (b) model, (c) metformin, (d) AME(L), (e) AME(H).

In the blank group, the structure of the colon was normal, with closely arranged epithelial cells of the mucosa that showed no shedding or decrease. The mucosa had an intact structure and a uniform thickness of the muscular layer, with no inflammatory infiltration. In the model group, the villus structure of the mucosa was abnormal, exhibiting discordant length, loose arrangement, swelling, and a decrease in number. Inflammatory infiltration was observed in the mucosa. In the low‐dose AME group, the villi were orderly arranged with relatively consistent length and a small amount of inflammatory infiltration. The colon structure in the high‐dose AME group was normal, and the villi were also orderly arranged. The length of the colon in the high‐dose AME group was similar to that in the blank group (Figure 6).

The pathogenesis of diabetes remains unclear. This study investigated the use of AME as a treatment strategy for diabetes. Our findings demonstrate that AME effectively lowers blood glucose and blood lipids in diabetic rats, while also mitigating changes in liver histopathology. Furthermore, AME improves the abnormal structure of colon tissue and regulates the imbalance of intestinal flora. In conclusion, our results suggest that AMEs may have a therapeutic effect in slowing the progression of diabetes.

In this study, we established a T2D model by using STZ injection and a high‐fat diet. As expected, we observed several significant pathophysiological changes in diabetic rats, including increased blood glucose and lipids. These findings are consistent with previous studies (Liu et al., 2023; Zhang et al., 2023). Interestingly, after administering AME, we observed a statistically significant decrease in blood glucose and lipid levels. Furthermore, we found evidence of liver damage in T2D rats, which aligns with previous pathological findings in T2D (Xu et al., 2023). Encouragingly, the administration of AME improved the aforementioned pathological changes.

The literature has increasingly shown that STZ‐induced diabetes can result in intestinal dysbacteriosis and structural changes in the intestines. For instance, a recent study demonstrated that dysregulation of the gut microbiota contributes to the development of T2D, and modulating the structure and diversity of the gut microbiota could be a potential therapeutic approach to improve T2D (Adeshirlarijaney & Gewirtz, 2020). Building upon these findings, our study revealed significant alterations in microbial community richness and diversity in the gut of diabetic rats after destruction, with a notable difference between the populations, favoring the control group. Furthermore, our study found that the effects of AME administration on the α‐diversity of intestinal flora were similar to other interventions reported in the literature. For example, dietary interventions involving butyrate and fiber did not impact microbial α‐diversity but did alter its composition (Huang et al., 2023; So et al., 2018; Yang et al., 2018). These findings suggest that certain interventions do not induce changes in the α‐diversity of the gut microbiota. Additionally, to investigate which intestinal flora is regulated by AMEs, we conducted LEfSe analysis and observed differences in the relative abundance of representative intestinal flora between diabetic rats and controls. A similar phenomenon was observed in another study. Moreover, some representative components of the intestinal microflora in the AME group overlapped with those in the control group, indicating that the AME group could partially restore the changes in intestinal microflora and reconstruct a healthy intestinal microenvironment.

Our findings have potential therapeutic implications. Our animal studies have shown that the administration of AME not only regulates the intestinal flora but also has a potential effect on diabetes, making it a potential treatment for diabetes. However, it is important to note that the study has several limitations. First, each group of mice had a small sample size, which may impact the generalizability of the results. Additionally, the dynamic changes of intestinal flora could not be observed by collecting intestinal content samples only at the end of the experiment. To address these limitations and further validate the findings of this study, it is recommended to conduct more extensive animal model samples, include more time points, and perform more detailed molecular biology experiments.

This study aimed to investigate the effect of AME therapy on the induction of high‐fat diet (HFD) in STZ T2D rats and its protective effect on abnormal glucose and lipid metabolism. The findings revealed that AME was able to regulate the intestinal microflora composition in T2D rats and enhance the abnormal structure of intestinal tissue, ultimately delaying the progression of abnormal glucose and lipid metabolism in T2D. These results lay the groundwork for further investigation into the mechanisms behind delaying abnormal glucose and lipid metabolism in T2D, and suggest a novel approach for the management of glucose and lipid metabolism disorders in diabetes mellitus through the use of AME.

## AUTHOR CONTRIBUTIONS

Dan Song: Data curation (equal); formal analysis (lead); validation (lead); visualization (lead); writing–original draft (equal). Cheng Fang: Conceptualization (lead); funding acquisition (lead); methodology (equal); project administration (equal); resources (lead); supervision (equal); writing–original draft (equal); writing–review and editing (lead).

## CONFLICT OF INTEREST STATEMENT

The authors declare that they have no conflict of interest.

## ETHICS STATEMENT

The Animal Experience Ethics Committee of Heilongjiang University of Chinese Medicine has reviewed and granted approval for the animal study.

## Data Availability

The data that support the findings of this study are available on request from the corresponding author. The data are not publicly available due to privacy restrictions.
